# Lossless and Near-Lossless Image Compression Using Generalized Multi-Context Linear and Nonlinear Prediction

**DOI:** 10.3390/e28080838

**Published:** 2026-07-27

**Authors:** Grzegorz Ulacha, Mirosław Łazoryszczak

**Affiliations:** Faculty of Computer Science and Information Technology, West Pomeranian University of Technology in Szczecin, ul. Żołnierska 49, 71-210 Szczecin, Poland

**Keywords:** entropy coding, lossless image coding, linear prediction, near-lossless compression

## Abstract

The paper proposes the Multi-ctx2 method, which enables image compression in lossless and near-lossless modes. It employs a generalized multi-context division method in the prediction stage, which is more efficient than other fast prediction methods. Five simple rules for computing the context number have been developed, which serve not only to identify an individualized linear predictor but also to correct the cumulative prediction error. In subsequent stages of the encoder, prediction errors are encoded in a two-stage process: first using an adaptive Golomb code, then a binary adaptive arithmetic encoder. The proposed method is characterized by short compression and decompression times while offering a good compromise between compression efficiency and encoding/decoding time. The proposed prediction method can be easily implemented in hardware due to its use of fixed-point arithmetic. Unlike many other solutions, there is no need to access the entire image data during encoding to tune the encoder parameters to a specific image. The paper demonstrates the efficiency of the proposed solution compared to competing solutions, showing improvements of 6.85% and 7.03% over JPEG-LS in lossless mode (and 10.2% in near-lossless mode) across two test sets.

## 1. Introduction

In IT systems, optimizing operating costs plays a significant role. These costs can be reduced both during transmission (by limiting bandwidth) and during data archiving (by compressing data). This applies particularly to the storage requirements for multimedia data. Energy consumption is also a significant factor, both on the data archiving server and on mobile devices.

This paper focuses on lossless image compression, which among other things is useful for archiving 2D, 3D, and 4D (three-dimensional video sequences) medical images [1,2,3,4,5], astronomical images, and satellite imagery [6,7]. Remote detection is increasingly important with the development of lightweight drones and small satellites that capture various types of imagery, such as multispectral imagery; in this context, lossless mode is often required. Furthermore, lossless compression is often required during the graphic processing of photos and advertising materials, as well as in the production of television programs, films (post-production [8]), etc.

Modern multimedia data compression typically involves two stages: data decomposition to reduce redundancy, and compression using efficient entropy-based methods, with arithmetic and Huffman coding being among the most efficient [9]. For images, decomposition significantly reduces redundancy caused by high correlation between neighboring pixels. At this stage, the most widely used methods are the predictive JPEG-LS [10] and CALIC methods [11], as well as the wavelet-based JPEG2000 method [12]. Most studies employ linear or nonlinear prediction. Information on various approaches to lossless coding can be found in review articles [13,14,15].

The most efficient lossless compression methods are those with high implementation complexity and belong to the class of time-symmetric methods (where the decoding time equals the encoding time). In such cases, the methods are based on linear prediction models with backward adaptation using mechanisms known from the literature, such as RLS [16], OLS [17,18,19], or WLS [20,21], in which the encoding and decoding of each subsequent pixel are accompanied by a procedure for adapting or re-estimating the linear predictor coefficients. The latest deep learning and machine learning solutions make full use of nonlinear neural networks [22]. They are also typically characterized by high computational complexity, and their short (though not in all cases) encoding/decoding times are achieved solely through high parallelization while utilizing GPU/NPU technologies that consume significant energy. Therefore, this paper proposes a relatively efficient method that offers short encoding/decoding times without the need for highly efficient dedicated computing units.

Section 2 discusses the fundamentals of image modeling that enable efficient compression in lossless and near-lossless modes by reducing data redundancy. Examples from the literature are used in reviewing those methods of predictive modeling with multi-context partitioning on which the proposed method is based. This method is presented in detail in Section 3, where its high compression efficiency in both lossless and near-lossless modes is also demonstrated through experimental results. The effectiveness of the proposed method stems from well-chosen simple decision rules for computing the context number and from pretraining predictive models for specific contexts, a similar approach to that used in deep neural networks.

## 2. Basics of Image Modeling

**Figure 1:**
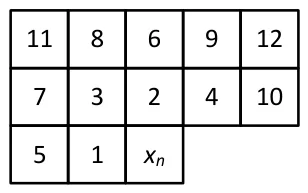
Numbering of neighborhood pixels relative to the currently encoded pixel $x_{n}$.

One approach to removing cross-correlation in encoded images is to use linear prediction with an appropriate selection of neighboring pixels and a prediction order. Due to the image encoding direction assumed in this work (successive rows are encoded from top to bottom and each row from left to right), both the encoder and the decoder have access to the pixels above and to the left of the currently encoded (decoded) pixel; we refer to this as the principle of causality. Assuming that correlation decreases with increasing distance between pixels, we can number the neighbors of the encoded pixel by increasing Euclidean distance $\sqrt{{\left(\Delta x_{j}\right)}^{2}+{\left(\Delta y_{j}\right)}^{2}}$ between their centers. Pixels at the same distance are numbered in a clockwise direction. This results in a one-dimensional signal domain, which simplifies the mathematical notation of many formulas and relationships known from the literature for one-dimensional signals. Figure 1 illustrates the twelve numbered nearest neighbors (used in the proposed solution) of the encoded pixel $x_{n}$, where the *j*th number indicates a pixel with value P(j). Theoretically, the higher the pixel number, the less significant it is for improving coding efficiency.

Various techniques are used for modeling, though a typical linear predictor of order *r* is most commonly used in lossless image compression; this predictor gives the predicted value of the pixel $x_{n}$ currently being encoded based on *r* neighboring pixels in accordance with the causal relationships known to the encoder and decoder. The linear predictor is of the following form:

(1) $${\hat{x}}_{n}=\sum_{j=1}^{r}b_{j}\;\cdot \;P\left(j\right)$$

where the elements of P(j) are the pixel values from the immediate neighborhood of the currently encoded pixel $x_{n}$, while the elements of $b_{j}$ are the prediction coefficients that form the vector **B** [9]. In practical applications, it is often assumed that the sum of the coefficients in such a model should equal 1 (this is the condition for an unbiased prediction estimator). Under this assumption and with an 8-bit grayscale representation of the input data, the predicted value ${\hat{x}}_{n}\in \left[0,255\right]$. Using a linear or nonlinear predictor permits encoding only the prediction errors, i.e., the differences $e_{n}$ between the actual and predicted values (rounded to the nearest integer), which are usually small and oscillate near zero:

(2) $$e_{n}=x_{n}-\left[{\hat{x}}_{n}\right].$$

In this way, we obtain a differential image in which the distribution of errors $e_{n}$ is close to a geometric distribution, enabling efficient encoding with one of the entropy-based methods. A higher compression ratio can be achieved by using a near-lossless mode, which allows us to define the maximum permissible absolute value *d* of the difference in pixel values between the original and decoded images. This approach is often used to store images in a visually lossless manner, with the acceptable error level determined by a group of experts. This mode is most often based on predictive coding, where the set of prediction errors $e_{n}$ is quantized as follows [23]:

(3) $${\hat{e}}_{n}=\left\{\begin{array}{cc} \left\lfloor \frac{e_{n}+d}{2d+1}\right\rfloor & \mathrm{if}\;e_{n}\geq 0,\\ \left\lfloor \frac{e_{n}-d}{2d+1}\right\rfloor & \mathrm{if}\;e_{n}<0. \end{array}\right.$$

We can reconstruct the original prediction error with accuracy ±d, yielding

(4) $${\tilde{e}}_{n}={\hat{e}}_{n}\;\cdot \;\left(2d+1\right).$$

When d=0, we obtain a lossless mode.

### 2.1. Fixed Predictive Models

A fixed predictor has a fixed set of prediction coefficients and can efficiently encode images with different characteristics. In the simplest solutions, the number of neighboring pixels determines the prediction range, which for a universal predictor (not associated with a specific image) should not be too large; hence, most fixed predictors discussed in the literature use only three to four pixels from the immediate neighborhood. This rule does not apply, for example, to customized predictors, in which coefficients are determined for individual images, e.g., by minimizing mean-squared error or absolute error [24].

The vast majority of fixed-point predictors proposed in the literature have coefficients that are powers of two (or sums of a small number of such powers, e.g., 0.625=0.5+0.125). Fixed-point calculations facilitate the hardware implementation of the predicted value, enabling low-power algorithm. Multiplication can then be replaced by addition, subtraction, and bit shifts, as described in [25], from which predictors Kuroki6, Kuroki7, Kuroki9, and Kuroki10 are derived. An exception among those examined here is the set of predictors presented in [26], for which the coefficients, rounded to two decimal places, were also used in this work (under the names TLR85, Plane3, Daab1, Daab2, Daab5, Daab8). The basic and most frequently used set of predictors consists of four adjacent pixels P(1), P(2), P(3), P(4) (see Figure 1) as well as their linear combinations JPEG5, JPEG6, JPEG7 proposed in the first version of the JPEG lossless codec [9,27]. Predictor FIR1 comes from [28], GradNorth, GradWest, Pirsh, Mean-4, Plane, and Plane2 from [29], and Deng10, Deng11, Deng12, Deng13 from [30].

Table 1 presents coefficients for 30 fixed prediction models obtained from the studies mentioned above. Each of them was subjected to a testing procedure that calculated the average prediction error entropy based on measurements from 45 test images, allowing them to be ranked by efficiency (from those with the statistically lowest prediction error entropy).

### 2.2. Block-Based Predictive Modeling Methods

By applying the principle of area diversity within a single image, the image can be divided into blocks (e.g., 8×8 or 16×16 pixels). Each block is assigned a separate predictive model. One of the earliest approaches of this type was presented in [31], in which each 8×8-pixel block was assigned one of eight fixed models that produced the smallest absolute error. The header information for this block required three bits to identify the predictive model number. Subsequent developments introduced minimizing the mean square error as a criterion for selecting the best set of prediction coefficients; however, this led to a very large header because storing the prediction coefficients required many bits. To reduce the header size, blocks with similar characteristics were grouped into clusters, each associated with a common prediction model [32]. Vector quantization techniques (as well as fuzzy clustering [33]) were used to create optimized sets, e.g., 16 predictive models, so that even with a large number of predictions, the total header size did not significantly increase the average bit rate. In ref. [34], adjacent blocks belonging to the same category (associated with the same predictor) were combined into groups, creating larger blocks. Subsequently, a map of these blocks of varying sizes was encoded using an efficient quad-tree coding technique. The main drawback of this vector quantization approach is the high encoder complexity, which, for example, in the case of the MRP 0.5 codec [34], results in encoding times of minutes for a 512×512 pixel image. It is straightforward to show that prediction coefficients can be obtained that yield lower entropy than those from the MMSE method, as demonstrated, for example, in [34]. In ref. [35], the authors proposed minimizing theMinimum Mean Absolute Error (MMAE), which yielded better results than MMSE in the case of block partitioning. A more detailed justification of the suboptimal impact of MMSE on entropy is presented in [36]. Therefore, in our solution we use the convenient IRLS method for fast estimation of prediction coefficients by minimizing absolute error, as discussed in detail in [24].

### 2.3. Adaptive Prediction with a Switchable Model

An alternative to dividing an image into blocks is context partitioning. In this approach, the implementation complexity of both the encoder and the decoder is relatively low, and it is not necessary to provide the decoder with header data in the form of prediction coefficient sets. We define context as a set of features characterizing the immediate surroundings of the encoded pixel. By using contextual partitioning (at the level of a single pixel rather than an entire block) and detecting different types of neighborhoods (classes with distinct features), we can tailor linear predictive models to each neighborhood’s characteristics, thereby improving compression efficiency. Because of the context switching mechanism, such predictors are sometimes referred to as nonlinear or multichannel predictive coders. The efficiency of such solutions surpasses that of the universal Plane3 predictor, which ranked first among the 30 fixed predictors listed in Table 1. An example is the Median Adaptive Predictor (MAP) proposed in the JPEG-LS algorithm, which is based on the Median Edge Detector (MED) context switching technique [10,37,38]. It uses a set of three simple predictors with context switching. The context number is determined by three adjacent pixels: P(1), P(2), and P(3). The predicted value is calculated using the following rule:

(5) $${\hat{x}}_{MED}=\left\{\begin{array}{cc} \min(P(1),P(2)) & \mathrm{if}\;P(3)\geq \;\max(P(1),P(2))\;\\ \max(P(1),P(2)) & \mathrm{if}\;P(3)\leq \;\min(P(1),P(2))\; \end{array}\right.$$

and ${\hat{x}}_{MED}=P\left(1\right)+P\left(2\right)-P\left(3\right)$ in all other cases. If we translate the above rule into Algorithm 1 for context switching, we obtain a method for selecting one of three contexts, each associated with a fixed linear predictor.
**Algorithm 1** Calculating the context number in the MED method.  1:**if** (P(1)>P(2)) **then**  2:      max = P(1);  3:      min = P(2);  4:**else**  5:      max = P(2);  6:      min = P(1);  7:**end if**  8:**if** (P(3)≥ max) **then**  9:      context = 1;10:**else if** (P(3)≤ min) **then**11:      context = 2;12:**else**13:      context = 3;14:**end if**

For context 1, the predicted value is given by ${\hat{x}}_{MED}=$ min(P(1),P(2)); for context 2, ${\hat{x}}_{MED}=$ max(P(1),P(2)); and for context 3, ${\hat{x}}_{MED}=P\left(1\right)+P\left(2\right)-P\left(3\right)$. Several extensions of this idea have been developed [39,40], but the resulting improvement was not significant compared with methods using a larger number of contexts, for which we will present the principles of operation in the following sections. For example, the MED_+_ algorithm described in [40] expands the number of contexts in MED to seven by introducing additional thresholds $t_{1}$ and $t_{2}$ that distinguish between small and large gradients in the immediate neighborhood of the encoded pixel.

#### 2.3.1. Gradient-Adjusted Predictor (GAP) Context-Based Prediction Method GAP_+_

The Gradient-Adjusted Predictor (GAP) context-based prediction method, which uses fixed coefficients for each model was proposed as a preliminary prediction method in the CALIC algorithm [11]. Increasing the number of contexts leads to more accurate predictions. Therefore, unlike the MED method described in the previous subsection, which uses three neighboring pixels to determine the predicted value and select one of three contexts, the GAP method uses seven neighboring pixels to determine the predicted value and allows selection of one of seven contexts. These are the pixels P(j) with j={1,2,3,4,5,6,9}. The appropriate context is selected based on the quantization of $d_{GAP}=d_{h}-d_{v}$, where $d_{h}$ and $d_{v}$ are the directional deviation levels (gradients) of the neighborhood [11]:

(6) $$\begin{array}{cc} d_{h}= & \left|P(1)-P(5)\right|+\left|P(2)-P(3)\right|+\left|P(4)-P(2)\right|,\\ d_{v}= & \left|P(1)-P(3)\right|+\left|P(2)-P(6)\right|+\left|P(4)-P(9)\right|. \end{array}$$

**Figure 2:**
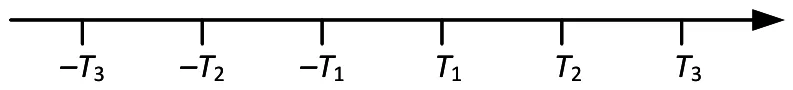
Seven-level scalar quantizer $x_{n}$.

The range of $d_{GAP}$ values is divided into seven intervals using three experimentally determined thresholds by the method’s authors (six-threshold scalar quantization, as shown in Figure 2). For 256-gray-scale images, the absolute values of the thresholds are $T_{1}=8$, $T_{2}=32$, and $T_{3}=80$ (research in [41] led to changes in these thresholds to $T_{1}=6$, $T_{2}=25$, $T_{3}=78$). Below is a pseudocode representation of Algorithm 2 for determining the context number [11].
**Algorithm 2** Calculating the context number in the GAP method.  1:**if** $d_{GAP}>T_{3}$ **then**  2:      context = 7;  3:**else if** $d_{GAP}<-T_{3}$ **then**  4:      context = 6;  5:**else**  6:      context = 1;  7:      **if** $d_{GAP}>T_{2}$ **then**  8:           context = 5;  9:      **else if** $d_{GAP}>T_{1}$ **then**10:           context = 4;11:      **else if** $d_{GAP}<-T_{2}$ **then**12:           context = 3;13:      **else if** $d_{GAP}<-T_{1}$ **then**14:           context = 2;15:      **end if**16:**end if**

Each context is assigned a linear predictor that uses between one and four neighboring pixels. Table 2 lists the prediction coefficients $b_{j}$ for each of the seven contexts, and the predicted value is computed using Equation (1).

A modified version of the GAP method was presented in [42]. It is characterized by slightly higher efficiency and will be denoted GAP_+_ in this paper. Each of the seven contexts has been assigned a linear predictor that uses one to five of the six neighboring pixels. Table 3 lists the prediction coefficients for each of the seven contexts (a seven-item dictionary of predictors).

There are also other GAP-based methods, including those that use contextual partitioning to improve the efficiency of genetic algorithms [43,44].

#### 2.3.2. Gradient-Based Selection and Weighting

The Gradient-Based Selection and Weighting Pixel Predictor method (GBSW) presented in [45] is based on four directional gradients aligned with the cardinal directions relative to the currently encoded pixel $x_{n}$: *w*, *n*, nw, and ne, computed similarly to the GAP method. Four values are specified:

(7) $$\begin{array}{cc} d_{w}= & (2\left|P(1)-P(5)\right|+2\left|P(2)-P(3)\right|+2\left|P(3)-P(7)\right|+2\left|P(2)-P(4)\right|\\ \; & +\left|P(6)-P(8)\right|+\left|P(6)-P(9)\right|)/10,\\ d_{n}= & (2\left|P(6)-P(2)\right|+2\left|P(1)-P(3)\right|+2\left|P(3)-P(8)\right|+2\left|P(4)-P(9)\right|\\ \; & +\left|P(5)-P(7)\right|+\left|P(7)-P(11)\right|)/10,\\ d_{nw}= & (2\left|P(1)-P(7)\right|+2\left|P(2)-P(8)\right|+\left|P(3)-P(11)\right|+\left|P(4)-P(6)\right|)/6,\\ d_{ne}= & (2\left|P(5)-P(3)\right|+2\left|P(2)-P(9)\right|+\left|P(1)-P(2)\right|+\left|P(3)-P(6)\right|)/6. \end{array}$$

These values are associated with the predictors P(1), P(2), P(3), and P(4), respectively. Next, the two gradients with the smallest values are selected from among these four; these become the weight coefficients of the predictive model, which is a linear combination of two of the four nearest neighbors. The weights are associated with the predictors to obtain the best predicted value (using the cross-multiplication method of weights and pixels). For example, if the two smallest values are $d_{w}$ and $d_{n}$, the predicted value is determined as follows using the northern pixel P(2) and the western pixel P(1):

(8) $${\hat{x}}_{n({GBSW}_{+})}=\frac{d_{w}^{2}\;\cdot \;P\left(2\right)+d_{n}^{2}\;\cdot \;P\left(1\right)}{d_{w}^{2}+d_{n}^{2}}.$$

When the denominator in Equation (8) is 0, the predicted value is obtained from the GAP_+_ model. This improved GBSW_+_ method proved significantly more efficient than the MED and GAP variants discussed earlier.

## 3. An Improved Multi-Context Method

In addition to the previously mentioned multi-context encoding methods (MED, GAP, GAP_+_), new proposals with varying numbers of contexts have continued to emerge over the years; examples include works from 2001–2006 [39,40,45,46,47] as well as slightly more recent ones [48,49,50,51,52,53]. These methods offer the low computational complexity of both the encoder and the decoder together with relatively high compression efficiency. A common feature is a relatively small number of contexts, and their efficiency is typically comparable to that of GAP_+_ and GBSW_+_.

Assuming that an increase in the number of contexts improves compression efficiency, the concept of contextual segmentation can be generalized by introducing a much larger number of contexts, each with its own predictive model. Due to its universality (the codec’s independence from the individual characteristics of the encoded image), experiments have shown that using the twelve nearest neighbors (see Figure 1) yields the best results while still maintaining low implementation complexity (for example, compare the 106 neighbors used in the Blend-28 method [54]).

Compared to the methods described in Section 2.3, our Multi-ctx2 method proposed in this section incorporates several improvements to objective function minimization and multi-context partitioning. The proposed solution involves two stages. First, to ensure that the method is considered universal for a relatively broad class of images, the preliminary training stage is based on a training image database, resulting in five sets of predictive models with 3456 or 4096 contexts. This stage uses the IRLS method for fast minimization of the absolute error [24]. The training stage needs to be performed only once and is similar to deep neural network training. The training image database should be as diverse as possible in terms of features (smooth, edge-rich, and textured image fragments). In our research, we used 55 training images, which combined with eight lossless rotation phases (every 90 degrees and mirror reflections) yielded a total of 440 training images. None of the test images used in Section 3.5 to evaluate performance were part of the training image set.

After the training process, each set consists of a group of 3456 or 4096 14th-order predictive models, which are used to predict values based on the obtained context number and the method used to determine it. Each coefficient is stored in a dictionary with a fractional precision of 10 bits.

The second stage of the method proposed here is the encoding process for a specific image (not part of the training dataset). In addition to fast encoding and decoding, the advantages of the Multi-ctx2 method include the ability to encode in online mode, which does not require knowledge of the entire image before encoding begins (unlike methods based on vector quantization [34] or those using complex, adaptive WLS techniques [20]).

### 3.1. Components of the Proposed Codec

**Figure 3:**
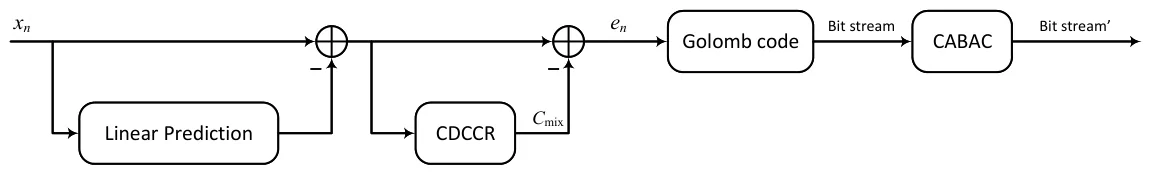
Block diagram of the cascade encoding scheme proposed in this paper.

The solution proposed in this paper is based on a cascaded approach (see Figure 3). In addition to the predicted value calculated using the authors’ context-dependent linear prediction, the Context-Dependent Constant Component Removing (CDCCR) block (similar to codecs such as CALIC or JPEG-LS) discussed in Section 3.4 is used to remove the constant component $C_{mix}$ associated with a specific context. The final blocks of the cascade are used to efficiently encode prediction errors $e_{n}$ using an adaptive Golomb encoder and a context-dependent adaptive binary arithmetic encoder.

The first block uses context-dependent linear prediction. When encoding each successive pixel, five context numbers (j={1,2,…,5}) are computed according to the rules in Section 3.1. Each number serves as an index into the corresponding table, which contains models with r=14 linear prediction coefficients $b_{\left(j,i\right)}$. The final predicted value is computed using the formula

(9) $${\hat{x}}_{n}=\frac{1}{5}\sum_{j=1}^{5}\sum_{i=1}^{14}b_{\left(j,i\right)}\;\cdot \;Z\left(i\right),$$

where the vector $Z=\left[z_{1},z_{2},\ldots ,z_{14}\right]=\left[{\hat{x}}_{n({GBSW}_{+})},{\hat{x}}_{n({GAP}_{+})},P\left(1\right),P\left(2\right),\ldots ,P\left(12\right)\right]$ contains the values of the twelve nearest neighbors of pixel $x_{n}$ (see Figure 1) as well as the two predicted values ${\hat{x}}_{n({GBSW}_{+})}$ and ${\hat{x}}_{n({GAP}_{+})}$ determined as described in Section 2.3.1 and Section 2.3.2, respectively. Using Equation (9) to compute the predicted value has lower computational complexity than modern deep learning-based methods.

**Figure 4:**
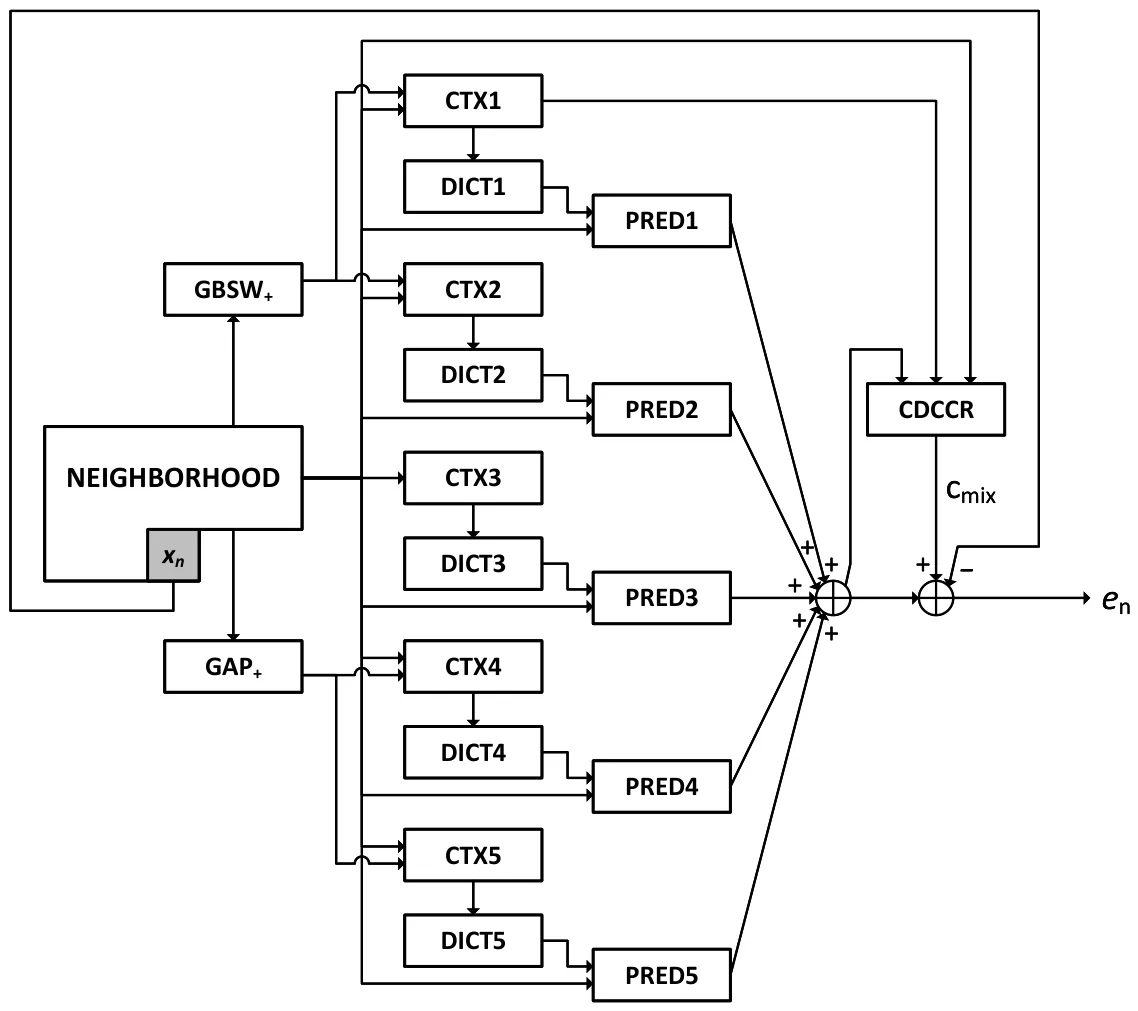
Block diagram of processing in linear prediction and CDCCR blocks.

The second data processing block removes the constant component associated with a given context (details are provided in Section 3.5). The data processing flowchart for the first two blocks is shown in Figure 4, where CTX*n* is the block for determining the *n*th context number, DICT*n* is the dictionary of prediction coefficients for the *n*th model, and PRED*n* is the block for calculating the value of the *n*th predictor. The last two blocks, shown in Figure 3 and used for encoding prediction errors, are described in detail in [54].

Algorithms 3 and 4, presented below, illustrate the data processing procedures in the encoder and decoder, respectively, of the solution proposed in this paper.
**Algorithm 3** The data processing process in the encoder.1:For each encoded pixel $x_{n}$:2:Determine numbers of the 5 types of contexts according to the rules described in Section 3.3.3:Calculate the predicted value (Equation (9)) and the prediction error $e_{n}$ (Equation (12)) after accounting for the context-dependent constant $C_{mix}$.4:Convert the prediction error $e_{n}$ into a bitstream using the adaptive Golomb coder.5:Encode the bit sequence from step 4 using an adaptive binary arithmetic encoder.6:Return to step 2 if there are still pixels to encode.

**Algorithm 4** The data processing process in the decoder.
1:For each decoded pixel $x_{n}$:2:Decode the input bit sequence using an adaptive binary arithmetic coder to obtain a Golomb code word.3:Convert the Golomb words to the form of prediction error $e_{n}$.4:Determine numbers of the 5 types of contexts according to the rules described in Section 3.3.5:Calculate the predicted value (using Equation (9)) and $C_{mix}$, and then add these values to $e_{n}$ to obtain the decoded pixel value $x_{n}$.6:Return to step 2 if there are still pixels to decode.


It is worth noting that the solution proposed here can also be used in a near-lossless mode by setting a maximum allowable error *d*, i.e., the difference between the original color value and the decoded value.

### 3.2. Context Segmentation Main Function Set

The proposed solution uses three methods to determine the context number, two of which are applied twice by substituting different values for the parameter representing the initial predicted value ${\hat{x}}_{n}$ of the currently encoded pixel $x_{n}$. Two simple methods for determining the predicted value were used for this purpose, namely, the GAP_+_ method (see Section 2.3.1) and the GBSW_+_ method (see Section 2.3.2), yielding five different predicted values for the currently encoded pixel $x_{n}$. Their arithmetical mean serves as the basis for the formula for determining the final predicted value.

By analogy with neural networks, the prediction method in Multi-ctx2 can be viewed as a simplified MLP network with a single hidden layer. At the input, we have twelve neighborhood pixels and two predicted values: GAP_+_ and GBSW_+_ (input layer). The hidden layer comprises five methods for determining the predicted value. The output layer consists of a single neuron that aggregates values from the hidden layer using an arithmetical mean (activation function ReLU, similar to hidden layer). Unlike MLP, in Multi-ctx2 it is possible to perform simple calculations in fixed-point format and omit the exponential function, which is a practical advantage for hardware implementation.

The context number is generated by combining decisions from various scalar quantizers (two-state or multi-state) based on the immediate neighborhood of the currently encoded pixel $x_{n}$. In this way, we obtain a set of features from the nearest neighborhood viewed from multiple perspectives, including the dependencies of the pixel $x_{n}$ surroundings relative to the initial predicted value ${\hat{x}}_{n}$. This is an input parameter, and two possibilities are considered here: ${\hat{x}}_{n}={\hat{x}}_{n(GBSW_{+})}$, used when calculating contexts 1 and 2, or ${\hat{x}}_{n}={\hat{x}}_{n(GAP_{+})}$, used when calculating contexts 4 and 5. Most decision rules are based on two-state quantizers that return a bit value of 0 or 1 (individual bits are denoted here as $\alpha_{i}$), indicating whether a specific condition is satisfied (YES/NO). Since the decision rules partially overlap across the different methods of constructing the context number, they are listed collectively here:

- $\alpha_{i}$—condition $P\left(i\right)>{\hat{x}}_{n}$ for $i=\left\{1,2,\ldots ,6\right\}$
- $\alpha_{7}$—condition $2P\left(1\right)-P\left(5\right)>{\hat{x}}_{n}$
- $\alpha_{8}$—condition $2P\left(2\right)-P\left(6\right)>{\hat{x}}_{n}$
- $\alpha_{i+8}$—condition $d_{i}>0$, where:
  (10) $$\begin{array}{cc} d_{1}= & \left|P(1)-P(5)\right|+\left|P(2)-P(3)\right|+\left|P(2)-P(4)\right|-\left|P(6)-P(2)\right|\\ \; & -\left|P(1)-P(3)\right|-\left|P(4)-P(9)\right|)\\ d_{2}= & 2\left|P(1)-P(7)\right|+2\left|P(2)-P(8)\right|+\left|P(3)-P(11)\right|+\left|P(4)-P(6)\right|\\ \; & -2\left|P(5)-P(3)\right|-2\left|P(2)-P(9)\right|-\left|P(1)-P(2)\right|-\left|P(3)-P(6)\right| \end{array}$$
- $\alpha_{11}$—condition $\left|P(1)-P(5)\right|>7$
- $\alpha_{12}$—condition $\left|P(2)-P(6)\right|>7$.

### 3.3. Setting the Context Number

This section discusses methods for determining the five types of contexts. The context number for type 1 is largely based on the nearest-neighbor relationship with respect to ${\hat{x}}_{n}={\hat{x}}_{n(GBSW_{+})}$. It is a 12-bit number of the form $\kappa_{11}\kappa_{10}\kappa_{9}\kappa_{8}\kappa_{7}\kappa_{6}\kappa_{5}\kappa_{4}\kappa_{3}\kappa_{2}\kappa_{1}\kappa_{0}$, where $\kappa_{i-1}=\alpha_{i}$ for i={1,2,…,10}. The last two bits, $\kappa_{11}\kappa_{10}$, are computed using a four-level quantizer for the variance ${\hat{\sigma }}^{2}$ with three thresholds {140,1100,9000}, where

(11) $${\hat{\sigma }}^{2}=\sum_{i=1}^{10}{\left({\hat{x}}_{n}-V\left(i\right)\right)}^{2}$$

and $V=[P\left(1\right),P\left(2\right),P\left(3\right),P\left(4\right),P\left(5\right),P\left(6\right),2P\left(1\right)-P\left(5\right),2P\left(2\right)-P\left(6\right),0.0625\;\cdot \;d_{1},$$0.125\;\cdot \;d_{2}]$. Context 4 is defined in exactly the same way but with ${\hat{x}}_{n}={\hat{x}}_{n(GAP_{+})}$ instead of ${\hat{x}}_{n}={\hat{x}}_{n(GBSW_{+})}$.

The context number of type 2 is a 12-bit number of the form $\kappa_{11}\kappa_{10}\kappa_{9}\kappa_{8}\kappa_{7}\kappa_{6}\kappa_{5}\kappa_{4}\kappa_{3}\kappa_{2}\kappa_{1}\kappa_{0}$, where the first eight bits are as follows: $\kappa_{i-1}=\alpha_{i}$ for i={1,2,…,5} and $\kappa_{i}=\alpha_{i+2}$ for i=5,6,7. The last four bits are computed from four binary quantizers, with value 1 (or 0) when the condition $\left|P\left(i\right)-{\hat{x}}_{n}\right|>7$ is satisfied (or not satisfied) for i={1,2,3,4}. Type 5 context is determined identically but using ${\hat{x}}_{n}={\hat{x}}_{n(GAP_{+})}$ instead of ${\hat{x}}_{n}={\hat{x}}_{n(GBSW_{+})}$ used in the case of type 2 context.

For contexts 1 and 2 (and their type 4 and 5 counterparts), there are $2^{12}=4096$ distinct context numbers; in contrast, context type 3 allows the context number to be chosen from 0 to 3455.

The first step in determining the number of the context type 3 is to calculate the three differences $d_{3}=P\left(1\right)-P\left(3\right)$, $d_{4}=P\left(1\right)-P\left(2\right)$, $d_{5}=P\left(4\right)-P\left(2\right)$. We quantize each of them (using a six-state quantizer that returns the interval number as an integer from 0 to 5) with the thresholds {−18,−5,0,5,18}, which correspond to the quantized value of $d_{i}$ labeled as follows: large negative, medium negative, small negative, small positive, medium positive, and large positive. With three integers from the range 0 to 5, we obtain $6^{3}=216$ possible states, which when combined with the four-bit number $\kappa_{3}\kappa_{2}\kappa_{1}\kappa_{0}=\alpha_{12}\alpha_{11}\alpha_{10}\alpha_{9}$ gives a total of $6^{3}\;\cdot \;2^{4}=3456$ numbers of the third context type.

### 3.4. Method for Correcting Cumulative Prediction Errors

In many cases, prediction methods may exhibit a constant component $C_{mix}$ in the calculated errors, the nature of which depends on the characteristics of the given context. For this reason, many solutions propose using an adaptive method to remove the constant component (bias cancellation), also known as context-specific prediction error correction, thereby improving the efficiency of predictive modeling.

Adaptive methods for removing the constant component are used in CALIC and JPEG-LS, among others. For each context, the number of occurrences and the cumulative sum of prediction errors are tracked in real time and the current estimate of the prediction error is corrected based on these values [11]. The proposed solution employs a similar approach, with the context number determined as in the type-1 context described in Section 3.2, except that to reduce the number of contexts to 2048, we do not use the most significant bit $\kappa_{11}$. Additionally, for the smallest images with no more than $2^{16}$ pixels, we reduce the number of contexts to 1024 (also omitting the $\kappa_{10}$ bit). This is because the cumulative prediction error is calculated adaptively for each *i*th context, and a large number of contexts slows the calculation of the correct approximate value of $C_{mix}$. Initially, the counts $N_{i}$ of occurrences of the *i*th context are set to 4, while the sum of prediction errors $S_{i}$ is set to 0. During encoding, the cumulative sum of prediction errors for the *i*th context is updated for each subsequent pixel: $S_{i}:=S_{i}+e_{n}$, and the size $N_{i}$ of that context is incremented by 1. The value of the constant term $C_{mix}=S_{i}/N_{i}$ is added to the predicted value computed using Equation (9). After rounding the result to the nearest integer, we obtain the final prediction error:

(12) $$e_{n}=x_{n}-\left[{\hat{x}}_{n}+C_{mix}\right]$$

which undergoes two-stage encoding using the adaptive Golomb code followed by the context-dependent adaptive binary arithmetic code CABAC; this stage is described in detail in [54].

### 3.5. Efficiency Analysis of an Improved Multi-Context Method

In the proposed method, we use the arithmetical mean of five predictive models as the predicted value; each model is associated with one of the five context types. To demonstrate the efficiency of each of the five approaches, we calculate the average bit rate for a set of 45 test images [55] independent of the training image set (see Table 4) based on a single prediction model indicated by the *i*th context number assignment type (labeling the columns as *K-i* for i={1,2,…,5}). Additionally, the table includes results obtained using other fast prediction methods ($MED_{+}$, $GAP_{+}$, and $GBSW_{+}$) known from the literature, as described in Section 2.3. These were substituted into our Multi-ctx2 codec at the prediction-error estimation stage for the purposes of the experiment.

Table 5 compares the average bit rates of several well-known fast lossless image compression methods from the literature. The averaged results for a standard set of nine test images at a resolution of 720×576 show that the Multi-ctx2 method offers the lowest average bit rate, outperforming even the ALCM_+_ method, which uses a content-optimized linear predictor of order r=24. In turn, Table 6 presents results for several of the latest classical lossless image compression methods as well as those based on deep learning (LCIC, L3C, CWPLIC, LCIC duplex). The proposed solution demonstrated the lowest average bit rate in the vast majority of cases, with an average improvement over JPEG-LS of 6.85% and 7.03%, respectively, for both sets of test images in lossless mode. The results in these tables confirm the effectiveness of the proposed solution.

This also confirms the notion that among widely available non-deep learning codecs, the best results are achieved with JPEG-XL in high-efficiency mode (switch “–distance=0 –effort=9”) as opposed to its default settings. This mode requires longer encoding times than the default settings. The situation is similar for WebP, where the encoding time for a 512 × 512—pixel image is just 0.144 s with the default settings. However, if we run this codec in the highest-performance mode (with the switches “-lossless -m 6 -q 100 -quiet”), the encoding time increases to 4.058 s (all time measurements presented in this paper were performed using a i5 3.4 GHz processor). Therefore, Table 7 presents the results for these specific codecs, using a set of 45 test images. These include not only photos from digital cameras but also images representing other specialized categories. Both Table 7 and Table 8 include a comparison with the H.266 codec, which is designed for video stream compression and is implemented using the VTM reference software for VVC with a set of switches that enable lossless compression.

A detailed list of ten sample images from other categories is provided in Table 8. The images labeled Airfield and Aerial were acquired using methods typical of remote sensing, while the image labeled Earth belongs to the space imaging category. Other specific images include Seismic and Finger, which contain seismic data and fingerprints, respectively. The next two images, Shapes and Noisesqare, are computer-generated; the first has smooth tonal transitions, while the second was generated with a high level of added noise. Among the images in the natural photography category, the image labeled Barb was selected as a specific example of images containing certain textured areas. Recently, there has also been a need to archive photographs of human silhouettes without elaborate backgrounds for the purposes of customizable virtual dressing; this category is represented by the images Ronaldo and Lakers24. It was mainly due to these last two images in the list from Table 8 that Multi-ctx2 ended up in third place. Nevertheless, it outperformed WebP in seven out of ten cases and JPEG-XL in half of the cases. This highlights a drawback of single-pass solutions (without selecting from many compression algorithms) based on pretraining. The problem can be partially solved by further expanding the training image dataset. On the other hand, the proposed solution offers higher efficiency than the CNN 2023 [56] method based on a cellular neural network, where the pretraining process took two months and the encoding and decoding time for an image is more than twenty times longer than in the case of Multi-ctx2.

**Table 5:** Results of bit averages for a set of standard test images.

Image	JPEG-LS [57]	CALIC [18]	Blend-7 [29]	HBB [58]	Multi-ctx [59]	ALCM_+_ [60]	Multi-ctx2
Balloon	2.889	2.78	2.84	2.80	2.727	2.733	2.675
Barb	4.690	4.31	4.43	4.28	4.243	4.211	4.185
Barb2	4.684	4.46	4.57	4.48	4.421	4.401	4.386
Board	3.674	3.51	3.57	3.54	3.467	3.425	3.415
Boats	3.930	3.78	3.84	3.80	3.730	3.696	3.683
Girl	3.922	3.72	3.76	3.74	3.664	3.612	3.599
Gold	4.475	4.35	4.42	4.37	4.310	4.293	4.288
Hotel	4.378	4.18	4.29	4.27	4.171	4.169	4.128
Zelda	3.884	3.69	3.79	3.72	3.700	3.598	3.658
Average	4.058	3.864	3.946	3.889	3.826	3.793	3.780

**Table 6:** Results of bit averages for the second set of test images based on experiments in [22].

Image	BPG	PNG	LCIC	JPEG 2000	JPLEG-LS	JPEG-XL	FLIF	WebP	L3C	CWPLIC	LCIC Duplex	Multi-ctx2
Airplane	4.32	4.26	3.99	4.00	3.80	3.71	3.82	3.87	4.56	3.69	3.69	3.572
Barbara	5.06	5.22	4.61	4.61	4.70	4.40	4.56	4.55	5.44	4.35	4.36	4.187
Coastg.	5.70	5.06	4.82	4.83	4.86	4.73	4.93	4.81	5.82	4.80	4.83	4.576
Comic	6.15	5.84	5.63	5.65	5.30	5.07	5.50	5.45	6.60	4.83	4.83	4.800
Flowers	5.18	5.08	4.91	4.92	4.62	4.51	4.74	4.76	5.53	4.41	4.35	4.320
Goldhill	4.95	4.70	4.58	4.59	4.43	4.37	4.50	4.47	5.27	4.33	4.33	4.203
Lennagr.	4.54	4.61	4.31	4.31	4.24	4.16	4.28	4.14	4.95	4.13	4.08	3.969
Mandrill	6.61	6.23	6.11	6.11	6.04	5.98	6.14	5.89	6.97	5.95	5.89	5.766
Monarch	4.10	4.26	3.82	3.82	3.70	3.54	3.68	3.73	4.37	3.40	3.45	3.388
Pepper	4.77	4.90	4.63	4.63	4.51	4.48	4.58	4.50	5.38	4.67	4.38	4.299
Ppt3	2.20	2.35	2.41	2.41	2.04	1.84	1.87	2.06	3.71	2.14	2.07	1.735
Zebra	5.83	5.19	4.89	4.89	4.81	4.66	4.84	4.86	6.08	4.65	4.68	4.504
Average	4.951	4.808	4.559	4.564	4.421	4.288	4.453	4.424	5.390	4.279	4.245	4.110

**Table 7:** Measurement of bit averages for a set of 45 standard test images.

PNG	H.266	WebP	JPEG-XL	Multi-ctx2
4.719	4.505	4.216	4.123	4.037

**Table 8:** Measurement of bit averages for images across different categories.

Image	PNG	H.266	WebP	JPEG-XL	Multi-ctx2
Airfield	5.824	5.741	5.086	4.918	4.841
Aerial	5.414	5.079	5.075	4.725	4.556
Earth	3.369	3.243	2.933	2.832	2.796
Seismic	3.139	3.185	2.954	2.734	2.661
Finger	5.817	6.270	5.564	5.324	5.342
Shapes	1.177	0.951	1.015	0.708	0.973
Noisesquare	5.755	5.664	5.085	5.245	5.269
Barb	5.213	4.644	4.550	4.271	4.185
Ronaldo	2.779	5.037	2.510	2.236	3.980
Lakers24	2.684	5.358	2.486	2.041	3.534
Average	4.117	4.517	3.726	3.503	3.814

In addition, the test images from the “Customizable Virtual Dressing” category are characterized by an unusual probability distribution (a small number of colors, resulting from preprocessing the photos using dithering, which introduces slight artificial noise into what was originally a fairly smooth background). For this reason, combining the Golomb code with CABAC without initial parameter tuning was ineffective at compressing prediction errors (WebP and JPEG-XL in high-performance mode support such tuning, which comes at a significant additional cost in terms of increased encoding time).

The following tables pertain to the near-lossless mode. Table 9 compares average bit rates and PSNR values for several methods from the literature, using the Lennagrey image as an example. Table 10 compares the results of LOCO-I and an extension of the TMW method to the near-lossless mode [61] (which has high implementation complexity in the encoding stage) with the Multi-ctx2 method proposed in this work for permissible error values d=2 and d=10. A much broader comparison set was compiled for d=1 (see Table 11). On average, the proposed method produced files that were 10.2% shorter than JPEG-LS at the given error threshold.

**Table 9:** Comparison of bit averages and PSNR values for the Lennagrey image.

Method	d=0		d=1		d=3	
	BPP	PSNR	BPP	PSNR	BPP	PSNR
JPEG-LS	4.24	∞	2.72	49.90	2.09	45.15
CALIC	4.11	∞	2.59	49.89	1.95	45.16
Vavelet [62]	4.30	∞	2.77	49.89	2.12	45.17
Two-stage [63]	3.99	∞	2.47	49.90	1.89	45.15
Multi-ctx2	3.969	∞	2.458	49.89	1.824	45.17

**Table 10:** Comparison of bit averages for the acceptable error value $d=\left\{2,10\right\}$.

Image	d=2			d=10		
	LOCO-I v.0.90N	TMW (Mode 3)	Multi-ctx2	LOCO-I v.0.90N	TMW (Mode 3)	Multi-ctx2
Bridge256	3.49	3.38	3.290	1.73	1.63	1.547
Camera256	2.28	2.08	1.964	0.96	0.90	0.726
Couple256	1.82	1.60	1.536	0.86	0.70	0.503
Airplane	1.84	1.64	1.563	0.72	0.57	0.466
Baboon	3.72	3.49	3.468	1.91	1.68	1.645
Lennagrey	2.09	1.83	1.821	0.93	0.55	0.485
Peppers	2.29	2.09	2.086	0.93	0.64	0.541
Shapes	0.79	0.75	0.545	0.47	0.58	0.248
Balloon	1.242	0.90	0.824	0.49	0.32	0.149
Boats	1.902	1.65	1.608	0.78	0.66	0.496
Gold	2.333	2.19	2.138	0.99	0.81	0.695
Average	2.163	1.964	1.895	0.979	0.822	0.682

**Table 11:** Comparison of bit averages for the acceptable error value d=1.

Image	JPEG-LS [10]	Sunset [23]	BAROLTO [23]	ASBOSC [64]	CALIC [18]	Multi-ctx2
Balloon	1.465	1.45	1.48	1.457	1.461	1.346
Barb1	3.149	3.10	2.88	2.868	2.837	2.684
Barb2	3.174	3.17	3.06	3.012	2.986	2.892
Board	2.203	2.22	2.09	2.033	2.038	1.951
Boats	2.478	2.48	2.39	2.306	2.315	2.217
Girl	2.446	2.38	2.28	2.258	2.250	2.137
Gold	2.996	3.06	2.92	2.904	2.874	2.790
Hotel	2.873	2.94	2.75	2.719	2.697	2.615
Zelda	2.375	2.26	2.25	2.221	2.225	2.161
Average	2.573	2.56	2.46	2.420	2.409	2.310

## 4. Conclusions

This paper proposes the Multi-ctx2 method, which enables image compression in lossless and near-lossless modes. During the prediction stage, it employs a generalized multi-context partitioning scheme in which each context number is assigned an individual linear predictor of order r=14. These predictors are fixed (determined using the MMAE method on a training image database and independent of the encoded image’s content), enabling fast online encoding without the need for preliminary feature analysis of the entire image, as required by many competing solutions. During the encoding of each pixel, after calculating the five context numbers and the initial predicted value, a simple method for correcting the cumulative prediction error is also applied. The prediction errors prepared in this way are encoded in a two-stage process using the Golomb code and a context-dependent adaptive binary arithmetic encoder.

The Multi-ctx2 encoder and decoder both have relatively low computational complexity that is comparable to that of other fast methods. The encoding and decoding times for the Lennagrey image (512×512 pixels) for a non-optimized version of the Multi-ctx2 code are 0.307 s and 0.303 s, respectively. These times do not depend on the content of the encoded image but scale linearly with the number of pixels. This represents a 13.4% reduction in time compared to the original method described in [59], with a noticeable increase in compression efficiency. Although the ALCM_+_ method offers compression efficiency similar to Multi-ctx2 with a slightly shorter decoding time of 0.245 s, it requires significantly greater computational effort during encoding. This is confirmed by the encoding time of the Lennagrey image, which is 1.925 s. Although there are methods with a few percent higher compression efficiency, their encoding time is typically several orders of magnitude higher as well; therefore, the proposed method appears to offer a good compromise between compression efficiency and encoding/decoding time.

## Figures and Tables

**Table 1:** Coefficient set for 30 fixed predictors.

Predictor	$b_{1}$	$b_{2}$	$b_{3}$	$b_{4}$	$b_{5}$	$b_{6}$	$b_{7}$	$b_{8}$	$b_{9}$	$b_{10}$
Plane3	0.66	0.66	−0.32	0	0	0	0	0	0	0
Pirsh	0.5	0.25	0	0.25	0	0	0	0	0	0
FIR1(Deng)	0.875	0.75	−0.625	0	0	0	0	0	0	0
JPEG6	0.5	1	−0.5	0	0	0	0	0	0	0
Kuroki6	0.75	1	−0.75	0	0	0	0	0	0	0
JPEG7	0.5	0.5	0	0	0	0	0	0	0	0
Kuroki7	1	0.75	−0.75	0	0	0	0	0	0	0
Kuroki9	0.25	0.75	0	0	0	0	0	0	0	0
JPEG5	1	0.5	−0.5	0	0	0	0	0	0	0
Daab2	0.67	0.33	0	0	0	0	0	0	0	0
Mean-4	0.25	0.25	0.25	0.25	0	0	0	0	0	0
SK	0.625	0.125	0.125	0.125	0	0	0	0	0	0
Plane	1	1	−1	0	0	0	0	0	0	0
Kuroki10	0.75	0.25	0	0	0	0	0	0	0	0
TLR85	1	0	−0.25	0.25	0	0	0	0	0	0
Daab5	0.6	0.2	0.2	0	0	0	0	0	0	0
Daab8	0.2	0.2	0.2	0.2	0	0	0	0	0	0.2
P2	0	1	0	0	0	0	0	0	0	0
P1	1	0	0	0	0	0	0	0	0	0
Daab1	0.67	0	0.33	0	0	0	0	0	0	0
Deng10	0	0.5	0	0.5	0	0	0	0	0	0
Deng13	1	1	−0.5	0	0	0	−0.5	0	0	0
Deng12	1	−0.5	−0.5	1	0	0	0	0	0	0
Far	0	0	0	0	0.25	0.25	0.125	0.125	0.125	0.125
GradNorth	0	2	0	0	0	−1	0	0	0	0
P3	0	0	1	0	0	0	0	0	0	0
Plane2	1	−1	0	1	0	0	0	0	0	0
P4	0	0	0	1	0	0	0	0	0	0
GradWest	2	0	0	0	−1	0	0	0	0	0
Deng11	1	−1	1	0	0	0	0	0	0	0

**Table 2:** A set of prediction coefficients corresponding to the individual contexts of GAP method.

Coeff.	Context						
	1	2	3	4	5	6	7
$b_{1}$	1/2	5/8	3/4	3/8	1/4	1	0
$b_{2}$	1/2	3/8	1/4	5/8	3/4	0	1
$b_{3}$	−1/4	−3/16	−1/8	−3/16	−1/8	0	0
$b_{4}$	1/4	3/16	1/8	3/16	1/8	0	0

**Table 3:** A set of prediction coefficients corresponding to the individual contexts of $GAP_{+}$ method.

Coeff.	Context						
	1	2	3	4	5	6	7
$b_{1}$	1/2	7/8	5/4	3/8	1/4	2	0
$b_{2}$	1/2	3/8	1/4	7/8	5/4	0	2
$b_{3}$	−1/4	−3/16	−1/8	−3/16	−1/8	0	0
$b_{4}$	1/4	3/16	1/8	3/16	1/8	0	0
$b_{5}$	0	−1/4	−1/2	0	0	−1	0
$b_{6}$	0	0	0	−1/4	−1/2	0	−1

**Table 4:** Measurement of bit averages for individual context-based prediction methods across a set of 45 standard test images.

MED_+_	GAP_+_	GBSW_+_	K-1	K-2	K-3	K-4	K-5	Multi-ctx2
4.125	4.110	4.107	4.047	4.046	4.050	4.046	4.048	4.037

## Data Availability

The datasets for this study are available upon request from the corresponding authors.

## References

[1] Kassim A., Yan P., Lee W.S., Sengupta K. (2005). Motion compensated lossy-to-lossless compression of 4-D medical images using integer wavelet transforms. IEEE Trans. Inf. Technol. Biomed..

[2] Sanchez V., Nasiopoulos P., Abugharbieh R. (2008). Efficient 4D motion compensated lossless compression of dynamic volumetric medical image data. Proceedings of the 2008 IEEE International Conference on Acoustics, Speech and Signal Processing.

[3] Scharcanski J. (2006). Lossless and Near-Lossless Compression for Mammographic Digital Images. Proceedings of the 2006 International Conference on Image Processing.

[4] Ström J., Cosman P.C. (1997). Medical image compression with lossless regions of interest. Signal Process..

[5] Xie X., GuoLin L., Zhihua W. (2007). A Near-Lossless Image Compression Algorithm Suitable for Hardware Design in Wireless Endoscopy System. EURASIP J. Adv. Signal Process..

[6] Chen X., Canagarajah C.N., Vitulli R., Nunez-Yanez J.L., Woods R., Compton K., Bouganis C., Diniz P.C. (2008). Lossless Compression for Space Imagery in a Dynamically Reconfigurable Architecture. Proceedings of the Reconfigurable Computing: Architectures, Tools and Applications.

[7] (2020). Lossless Data Compression. Blue Book.

[8] Andriani S., Calvagno G., Erseghe T., Mian G., Durigon M., Rinaldo R., Knee M., Walland P., Koppetz M. (2004). Comparison of lossy to lossless compression techniques for digital cinema. Proceedings of the 2004 International Conference on Image Processing. ICIP′04.

[9] Sayood K. (2018). Introduction to Data Compression.

[10] Weinberger M., Seroussi G., Sapiro G. (2000). The LOCO-I lossless image compression algorithm: Principles and standardization into JPEG-LS. IEEE Trans. Image Process..

[11] Wu X., Memon N. (1996). CALIC—A Context Based Adaptive Lossless Image Codec. Proceedings of the 1996 IEEE International Conference on Acoustics, Speech, and Signal Processing Conference Proceedings.

[12] Marcellin M., Gormish M., Bilgin A., Boliek M. (2000). An overview of JPEG-2000. Proceedings of the Proceedings DCC 2000.

[13] Carpentieri B., Weinberger M., Seroussi G. (2000). Lossless Compression of Continuous-Tone Images. Proc. IEEE.

[14] Deng G. (2002). Transform domain LMS-based adaptive prediction for lossless image coding. Signal Process. Image Commun..

[15] Memon N., Sayood K. (1995). Lossless image compression: A comparative study. Proc. SPIE-Int. Soc. Opt. Eng..

[16] Ulacha G., Stasinski R. (2009). Context based lossless coder based on RLS predictor adaption scheme. Proceedings of the 2009 16th IEEE International Conference on Image Processing (ICIP).

[17] Wu X., Barthel E., Zhang W. (1998). Piecewise 2D autoregression for predictive image coding. Proceedings 1998 International Conference on Image Processing, ICIP98 (Cat. No. 98CB36269).

[18] Ye H., Deng G., Devlin J. (2000). Adaptive linear prediction for lossless coding of greyscale images. Proceedings 2000 International Conference on Image Processing (Cat. No. 00CH37101).

[19] Ye H., Deng G., Devlin J. (1999). Least squares approach for lossless image coding. Proceedings of the ISSPA ’99. Proceedings of the Fifth International Symposium on Signal Processing and Its Applications (IEEE Cat. No. 99EX359).

[20] Ulacha G., Stasinski R., Wernik C. (2020). Extended Multi WLS Method for Lossless Image Coding. Entropy.

[21] Ye H. (2002). A Study of Lossless Compression of Greyscale Images. Ph.D. Thesis.

[22] Rhee H., Jang Y.I., Kim S., Cho N.I. (2021). Lossless Image Compression by Joint Prediction of Pixel and Context Using Duplex Neural Networks. IEEE Access.

[23] Xue X., Gao W. (1999). Prediction based on backward adaptive recognition of local texture orientation and Poisson statistical model for lossless/near-lossless image compression. 1999 IEEE International Conference on Acoustics, Speech, and Signal Processing. Proceedings. ICASSP99 (Cat. No. 99CH36258).

[24] Ulacha G., Łazoryszczak M. (2024). Lossless Image Compression Using Context-Dependent Linear Prediction Based on Mean Absolute Error Minimization. Entropy.

[25] Kuroki Y., Ueshige Y., Ohta T. (2001). An estimation of the predictors implemented by shift operation, addition, and/or subtraction. Proceedings 2001 International Conference on Image Processing (Cat. No.01CH37205).

[26] Daaboul A. Local Prediction for Lossless Image Compression. Proceedings of the Prague Stringology Club Workshop.

[27] Drozdek A. (2001). Elements of Data Compression.

[28] Marusic S., Deng G. (1999). A neural network based adaptive non-linear lossless predictive coding technique. Proceedings of the ISSPA ’99, Proceedings of the Fifth International Symposium on Signal Processing and Its Applications (IEEE Cat. No.99EX359).

[29] Seemann T., Tischer P. (1997). Generalized locally adaptive DPCM. Department of Computer Science Technical Report CS97/301.

[30] Deng G., Ye H. (1999). Lossless image compression using adaptive predictor symbol mapping and context filtering. Proceedings 1999 International Conference on Image Processing (Cat. 99CH36348).

[31] Memon N., Sayood K. (1995). An asymmetric lossless image compression technique. Proceedings, International Conference on Image Processing.

[32] Golchin F., Paliwal K. (1997). Classified adaptive prediction and entropy coding for lossless coding of images. Proceedings of the International Conference on Image Processing.

[33] Aiazzi B., Alparone L., Baronti S. (2002). Near-lossless image compression by relaxation-labelled prediction. Signal Process..

[34] Matsuda I., Ozaki N., Umezu Y., Itoh S. (2005). Lossless coding using variable block-size adaptive prediction optimized for each image. Proceedings of the 2005 13th European Signal Processing Conference.

[35] Hashidume Y., Morikawa Y. (2007). Lossless image coding based on minimum mean absolute error predictors. Proceedings of the SICE Annual Conference 2007.

[36] Wang X., Wu X. (2007). On Design of Linear Minimum-Entropy Predictor. Proceedings of the 2007 IEEE 9th Workshop on Multimedia Signal Processing.

[37] Deng G., Ye H. (2003). A general framework for the second-level adaptive prediction. Proceedings of the 2003 IEEE International Conference on Acoustics, Speech, and Signal Processing, 2003. Proceedings. (ICASSP ’03).

[38] Memon N., Wu X. (1997). Recent Developments in Context-Based Predictive Techniques for Lossless Image Compression. Comput. J..

[39] Estrakh D., Mitchell H., Schaefer P., Mann Y., Peretz Y. (2001). “Soft” median adaptive predictor for lossless picture compression. Signal Process..

[40] Jiang J., Grecos C. (2002). Towards an improvement on prediction accuracy in JPEG-LS. Opt. Eng..

[41] Ulacha G., Stasinski R. On context-based predictive techniques for lossless image compression. Proceedings of the IWSSIP 2005—Proceedings of 12th International Workshop on Systems, Signals and Image Processing.

[42] Wang H., Zhang D. (2004). A linear edge model and its application in lossless image coding. Signal Process. Image Commun..

[43] Leon D., Balkir S., Sayood K. (2002). An evolvable predictor for lossless image compression. Proceedings of the 2002 IEEE International Symposium on Circuits and Systems, Proceedings (Cat. No. 02CH37353).

[44] Salami M., Iwata M., Higuchi T. Lossless image compression by evolvable hardware. Proceedings of the Fourth European Conference on Artificial Life.

[45] Knezovic J., Kovac M. (2003). Gradient based selective weighting of neighboring pixels for predictive lossless image coding. Proceedings of the 25th International Conference on Information Technology Interfaces, ITI 2003.

[46] Chang C.C., Chen G.I. (2003). Enhancement algorithm for nonlinear context-based predictors. Vis. Image Signal Process. IEE Proc..

[47] Itani A., Das M., Bebis G., Boyle R., Koracin D., Parvin B. (2005). Adaptive Switching Linear Predictor for Lossless Image Compression. Proceedings of the Advances in Visual Computing.

[48] Avramović A. (2011). Lossless compression of medical images based on gradient edge detection. Proceedings of the 2011 19th Telecommunications Forum (TELFOR) Proceedings of Papers.

[49] Seyed Danesh A., Moradi Rad R., Attar A. (2010). A novel predictor function for lossless image compression. Proceedings of the 2010 2nd International Conference on Advanced Computer Control.

[50] Karimi N., Samavi S., Shirani S. (2010). Lossless compression of high-throughput RNAi images. Proceedings of the 10th IEEE International Conference on Information Technology and Applications in Biomedicine.

[51] Rad R.M., Attar A., Shahbahrami A. (2013). A predictive algorithm for multimedia data compression. Multimed. Syst..

[52] Hsu H.C., Ding J.J., Lu D.Y. (2025). Improved Low Complexity Predictor for Block-Based Lossless Image Compression. Eng. Proc..

[53] Chen G., Lin M. (2024). Sample-Based Gradient Edge and Angular Prediction for VVC Lossless Intra-Coding. Appl. Sci..

[54] Frydrychowicz M., Ulacha G. (2024). Two-Stage Golomb—Context-Adaptive Binary Arithmetic Coders Coding in Lossless Image Compression. Adv. Sci. Technol. Res. J..

[55] Ulacha G. (2022). Dataset of 45 Images. https://kakit.zut.edu.pl/fileadmin/Test_Images.zip.

[56] Nakashima K., Kawai Y., Nakazawa R., Toda H., Aomori H., Otake T., Matsuda I., Itoh S. (2023). Lifting-based lossless image coding using cellular neural network predictors and context estimators optimized by adaptive differential evolution. Nonlinear Theory Its Appl. IEICE.

[57] Strutz T. Context-based adaptive linear prediction for Lossless Image Coding. Proceedings of the 4th International ITG Conference on Source and Channel Coding.

[58] Seemann T., Tischer P., Meyer B. History-Based Blending of Image Sub-Predictors. Proceedings of the International Picture Coding Symposium.

[59] Ulacha G., Stasinski R. A New Fast Multi-Context Method for Lossless Image Coding. Proceedings of the 2018 International Conference on Sensors, Signal and Image Processing.

[60] Ulacha G., Łazoryszczak M. (2023). Lossless Image Coding Using Non-MMSE Algorithms to Calculate Linear Prediction Coefficients. Entropy.

[61] Meyer B., Tischer P. (1998). Extending TMW for near lossless compression of greyscale images. Proceedings DCC ’98 Data Compression Conference (Cat. No. 98TB100225).

[62] Yea S., Pearlman W. (2006). A Wavelet-Based Two-Stage Near-Lossless Coder. IEEE Trans. Image Process..

[63] Jakhetiya V., Au O.C., Jaiswal S.P., Jia L., Mittal G. (2014). Symmetrical predictor structure based integrated lossy, near lossless/lossless coding of images. Proceedings of the 2014 IEEE International Symposium on Circuits and Systems (ISCAS).

[64] Iordache R., Tabus I., Astola J. (1998). Fixed-slope near-lossless context-based image compression. Proceedings 1998 International Conference on Image Processing, ICIP98 (Cat. No. 98CB36269).

